# Cross-sectional study on COVID-19 vaccine hesitancy and determinants in healthcare students: interdisciplinary trainings on vaccination are needed

**DOI:** 10.1186/s12909-022-03343-5

**Published:** 2022-04-20

**Authors:** Sylvain Gautier, Domitille Luyt, Benjamin Davido, Marie Herr, Thomas Cardot, Anne Rousseau, Djillali Annane, Elisabeth Delarocque-Astagneau, Loïc Josseran

**Affiliations:** 1 Faculty of Health Sciences Simone Veil, University of Versailles Saint-Quentin-en-Yvelines, Montigny-le-Bretonneux, France; 2 Hospital Department of Epidemiology and Public Health, Raymond Poincaré Hospital, GHU University of Paris Saclay, Assistance Publique – Hôpitaux de Paris, Garches, France; 3 Inserm U1018, CESP, Primary Care and Prevention team, University of Paris Saclay, University of Versailles Saint-Quentin-en-Yvelines, Montigny-le-Bretonneux, France; 4 Infectious diseases Department, Raymond Poincaré Hospital, GHU University of Paris Saclay, Assistance Publique – Hôpitaux de Paris, Garches, France; 5 Inserm U1018, CESP, Anti-Infective Evasion and Pharmacoepidemiology team, University of Paris Saclay, University of Versailles Saint-Quentin-en-Yvelines, Montigny-le-Bretonneux, France; 6 Midwifery Department, Versailles Saint-Quentin-en-Yvelines University, Montigny-le-Bretonneux, France; 7 Department of Obstetrics and Gynecology, Poissy-Saint-Germain en Laye Hospital, Poissy, France; 8 Inserm U1018, CESP, Clinical Epidemiology team, University of Paris Saclay, Université of Versailles Saint-Quentin-en-Yvelines, Montigny-le-Bretonneux, France; 9 FHU SEPSIS (Saclay and Paris Seine Nord Endeavour to PerSonalize Interventions for Sepsis), RHU RECORDS (Rapid rEcognition of CORticosteroiD resistant or sensitive Sepsis), Department of Intensive Care, Hôpital Raymond Poincaré (AP-HP), Laboratory of Infection & Inflammation - U1173, University Versailles Saint Quentin - University Paris Saclay, INSERM, 92380 Garches, France

**Keywords:** COVID-19, Vaccination, Education, medical, Universities

## Abstract

**Background:**

To ensure the success of COVID-19 vaccination, public authorities need to have the support of the entire population and build vaccine confidence. Identifying and understanding the determinants of vaccine acceptance is essential for conducting vaccine strategy. The aim was to estimate vaccine hesitancy among healthcare students in France and to investigate the associated factors.

**Methods:**

A web-based cross-sectional study was conducted in a large French University in greater Paris area, among 4927 healthcare students from the different training courses such as medicine studies, midwifery studies, physiotherapy studies, nurse studies and others health studies. The study was conducted between January 21 and February 8, 2021 based on a questionnaire including 25 single or multiple-choice questions, made using the free software *Limesurvey*. The link of the questionnaire was distributed to the students by the teachers and the student associations. The SAGE group definition of vaccine hesitancy was used. All estimates were weighted using the gender and training courses category of all healthcare students registered for the 2020–2021 year. Crude and adjusted weighted odds ratio (wOR) and 95% confidence interval (95%CI) were estimated using logistic regression.

**Results:**

A total of 1465 healthcare students answered. A proportion of 44.5% (95%CI = [41.7–47.3]) of them were considered as hesitant. Women were more hesitant (50.9, 95%CI = [48.0–53.9]) than men (21.6, 95%CI = [15.2–28.0]). Vaccine hesitancy was significantly associated with gender (wOR = 0.27, 95%CI = [0.18–0.39]) and training courses: medical students were less likely to be hesitant than students in the common and first year of several health studies (wOR = 0.48, 95%CI = [0.33–0.70]) while nursing students were more than 5 times more likely to be hesitant (wOR = 5.20, 95%CI = [3.71–7.28]). Students who did an internship during the epidemic (wOR = 0.53, 95%CI = [0.41–0.69]) and who downloaded the mobile contact-tracing mobile app “TousAntiCovid” (wOR = 0.34, 95%CI = [0.26–0.44]) were significantly less likely to be hesitant.

**Conclusions:**

Overall vaccine hesitancy among healthcare students was high, substantial differences were found between training courses. To reduce these disparities, interdisciplinary lectures on vaccines for all healthcare students may be implemented and evaluated.

**Supplementary Information:**

The online version contains supplementary material available at 10.1186/s12909-022-03343-5.

## Background

The vaccination against COVID-19 represents an important hope to defeat the disease. WHO considers that all people should have access to safe and effective COVID-19 vaccines as quickly as possible, starting with those at high risk of severe disease or death [1]. Supply difficulties forced most countries around the world to first prioritize access to these vaccines for specific groups of populations highly vulnerable to COVID-19. The Strategic Advisory Group of Experts on Immunization (SAGE) has published two essential documents to help determine which groups should be vaccinated as a priority [2, 3]. Along with those having comorbidities (hypertension, heart disease, diabetes …) or elderly people, healthcare workers were considered as top priority groups considering exposure. At the same time, healthcare students were not considered as a priority group although they are in contact with patients during their internship. In many countries, as immunization coverage of priority groups increases, new groups of population were being called upon to be vaccinated like young people and healthcare students as a part of them.

To ensure the success of vaccination, public authorities need to have the support of the population and build vaccine confidence. In this context, identifying and understanding the determinants of vaccine acceptance is essential for conducting vaccine strategy, developing campaigns to promote vaccination and convincing the most reluctant people [4]. This is all the more true in France where vaccine hesitancy was deemed to be high [5]. According to the SAGE Working Group, vaccine hesitancy is defined as a delay in acceptance or refusal of vaccines despite availability [6].

A study conducted in early 2021 of 1942 French working-age adults showed that 28.8% of respondents categorically refused COVID-19 vaccination. That vaccine hesitancy was associated with age with an inverted U-shaped relationship: the youngest and the oldest showed greater acceptance to COVID-19 vaccination [7]. Studies have also shown that acceptance of COVID-19 vaccination in the general population is socially determined [8]. Because of insufficient vaccination coverage in the French population, the government introduced a covid-health pass for access to cultural activities or restaurants, as it has been done in other European countries.

As young people, healthcare students are expected to be less hesitant, especially since as future healthcare professionals they should be particularly concerned about vaccine prevention [9] and patients protection. However, there are few studies that specifically focus on the vaccine hesitancy of students and healthcare students in particular. A survey of 237 college students at the University of Rhode Island, United States of America (USA), in November 2020 found that 50% of respondents said they wanted to receive a COVID-19 vaccine as soon as possible [10]. Another study of 248 dental students and 167 medical students in USA found that 45% of dental students and 23% of medical students were reluctant to receive a COVID-19 vaccine [11]. In Europe, studies covering the pre-mass-vaccination campaign period are scarce. In a cross-sectional survey conducted in both nurses and nursing students of five southern European countries, vaccine hesitancy among students was circa 60% [12]. However, these works did not cover the diversity of health training courses. In this context, it was necessary to document vaccine hesitancy among the different healthcare students. Therefore, the aim of this study was to estimate vaccine hesitancy among the different training courses of healthcare students in France and to investigate the associated factors in order to inform vaccine strategy and, if needed, to suggest options for rethinking training programs on vaccines preventable diseases at the University.

## Methods

### Data collection

We conducted a web-based cross-sectional study in a large French University in greater Paris area including the different training courses such as medicine studies, midwifery studies, physiotherapy studies, nurse studies and others health studies (including studies in occupational therapy, psychomotricity, pedicure-podology, medical electro-radiology manipulation and others). The online questionnaire was developed using the freeware *Limesurvey* and available online between January 21 and February 8, 2021. The questionnaire was pre-tested by several 4th year medical students to ensure comprehension and feasibility. These students were asked not to complete the questionnaire again during its release. This study relied on a non-probability sampling method: teachers of all training courses and students’ associations were asked to distribute the link of the pre-tested questionnaire to all the healthcare students. After providing informed consent on the initial screen, participants were invited to answer 25 single or multiple-choice questions including several conditional questions. The questionnaire (available on supplementary 1) included 4 questions about age, gender, curriculum, and current training courses followed by the participants, 10 about the history, exposures, and experience of the COVID-19 outbreak, 4 about vaccination in general and 6 about COVID-19 vaccination in particular including a question on vaccine intent for COVID-19 vaccines. Depending on the answer to this question, participants were asked about the reasons for which they intend to be vaccinated or, on the contrary, not to be vaccinated. The corresponding Cronbach alpha values to test the internal consistency were 0.93 and 0.75 respectively.

Considering the population surveyed (*n* = 4927) and an ideal random sampling strategy, the minimum sample size was 878 respondents with a margin of error of 3% and a level of confidence in the responses of 95% without any assumption on the estimated proportions.

### Statistical analysis

**Fig. 1:**
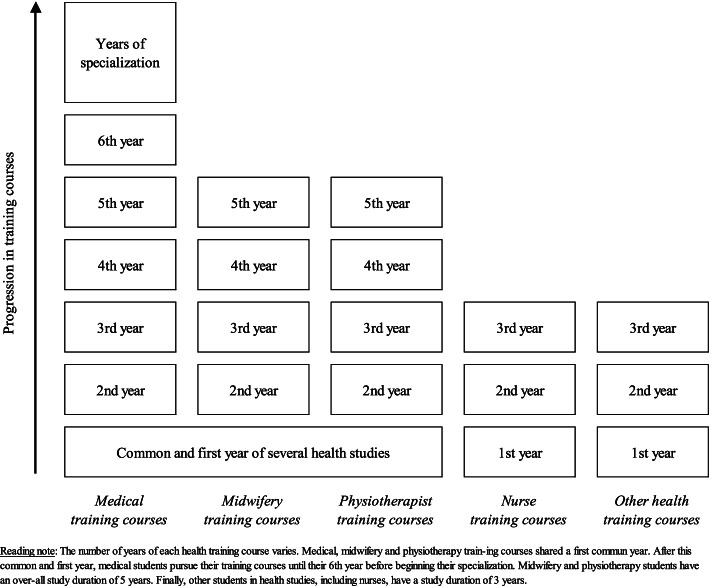
Description of the health training courses. COVID-19 vaccine hesitancy study among healthcare students, France, January–February 2021 (*n* = 1465)

Participants’ characteristics were described as numbers and percentages for all categorical variables and put next to the study population characteristics (Table 1). Considering the SAGE definition of vaccine hesitancy, we presented the vaccine hesitancy variable in 2 modalities instead of 5: “No hesitancy” (including “yes, definitely” and “yes, probably” previous modalities) and “Hesitancy” (including “no, certainly not”, “no, probably not” and “maybe” previous modalities). All population estimates were weighted: using the gender and training courses distribution of all the students registered for the 2020–2021 year, we calculated a posteriori weight for each responding student (Fig. 1). All variables are presented as unweighted numbers and weighted proportions in percent (Table 2).

**Table 1:** Characteristics of the participants and the study population. COVID-19 vaccine hesitancy study among healthcare students, France, January–February 2021 (*n* = 1465)

n (%)	Sample *N* = 1465	Study population^1^*N* = 4927
Gender		
Female	1219 (83.2)	3842 (78.0)
Male	246 (16.8)	1085 (22.0)
Age		
< 20 years	491 (33.5)	N.A.^2^
[20–22] years	623 (42.5)	
> 22 years	351 (24.0)	
Training courses		
Common and first year of several health studies	334 (22.8)	814 (16.5)
Medical studies (2nd year to 6th year)	349 (23.8)	745 (15.1)
Midwifery studies (2nd year to 5th year)	97 (6.6)	295 (6.0)
Physiotherapy studies (2nd year to 5th year)	174 (11.9)	952 (19.3)
Nursing studies (1st year to 3rd year)	342 (23.3)	1694 (34.4)
Other health studies^3^	169 (11.5)	427 (8.7)

^1^ All healthcare students of the Faculty of Health, University of Versailles Saint-Quentin-en-Yvelines

^2^ Not available

^3^ Including students in occupational therapy, psychomotricity, pedicure-podology, medical electro-radiology manipulation and others

**Table 2:** Opinions and attitudes of healthcare students towards COVID-19 vaccines and their history, exposures and experience of the COVID-19 outbreak. Unweighted numbers and weighted proportions in percent. COVID-19 vaccine hesitancy study among healthcare students, France, January–February 2021 (*n* = 1465)

n (%)^*^	Total ***N*** = 1465	Female ***N*** = 1219	Male ***N*** = 246
**Were you in internship during the epidemic**^†^**(y/n)**^‡^	1103 (97.8)	913 (97.2)	190 (99.7)
***If yes, did you take care of COVID-19 patients? (y/n)***	*670 (58.5)*	*550 (59.6)*	*120 (54.6)*
**Have you been infected with COVID-19?**			
Yes, with a positive test that proves it	199 (14.7)	165 (14.2)	34 (16.6)
Yes, I am convinced of it, but I did not perform a test	119 (8.4)	98 (8.3)	21 (8.7)
No/I do not know	1147 (76.9)	956 (77.5)	191 (74.7)
**Have there been any cases among your relatives? (y/n)**	1020 (68.1)	851 (68.6)	169 (66.3)
***If yes, were they hospitalized for COVID-19? (y/n)***	*188 (12.9)*	*167 (14.4)*	*21 (7.8)*
**Regarding your feelings since the beginning of the epidemic, which sentence(s) do you agree with?**			
I feel or have felt isolated	841 (57.2)	704 (57.0)	137 (57.9)
I am or was anxious	488 (34.4)	430 (36.8)	58 (25.4)
I am or was concerned about my health	218 (16.5)	197 (17.8)	21 (11.7)
I am or was concerned about my relatives’ health	1051 (74.9)	901 (76.1)	150 (70.1)
Following the courses online was difficult	968 (71.0)	818 (71.2)	150 (70.1)
**Have you been vaccinated against the flu this winter? (y/n)**	353 (26.7)	272 (25.3)	81 (74.7)
**Do you intend to be vaccinated against COVID-19?**			
*No hesitancy:*			
Yes, definitely	576 (36.7)	427 (31.6)	149 (54.9)
Yes, probably	286 (18.7)	231 (17.3)	55 (23.5)
*Hesitancy:*			
Maybe	257 (17.6)	242 (20.2)	18 (8.5)
No, probably not	184 (13.5)	171 (15.4)	13 (6.7)
No, certainly not	159 (13.5)	148 (15.5)	11 (6.4)
***If “No hesitancy” was answered: what are the reasons for being vaccinated against COVID-19?***			
*I want to protect myself against COVID-19*	*609 (70.8)*	*463 (71.2)*	*146 (69.7)*
*I want to protect my household members against COVID-19*	*802 (93.3)*	*614 (93.6)*	*188 (92.7)*
*I want to avoid COVID-19 transmission to patients*	*722 (84.6)*	*548 (83.9)*	*174 (86.2)*
*I want to be part of the epidemic control*	*694 (79.2)*	*527 (78.8)*	*167 (80.0)*
*I want to have my social, cultural, sporting interactions back*	*756 (88.4)*	*575 (88.3)*	*181 (88.7)*
*I believe that available vaccines are safe and efficient*	*466 (52.8)*	*330 (48.2)*	*136 (62.8)*
***If “Hesitancy” was answered: what are the reasons for being vaccinated against COVID-19?***			
*I prefer preventive measures or wearing mask to protect myself*	*196 (35.1)*	*185 (36.1)*	*11 (26.9)*
*I prefer to wait the safety of new vaccines demonstrated*	*456 (76.2)*	*428 (77.5)*	*28 (66.1)*
*I am afraid of side effects*	*319 (54.7)*	*298 (54.9)*	*21 (53.6)*
*I am against vaccination in general*	*20 (4.3)*	*19 (4.3)*	*1 (3.5)*
*I am not at risk-population of severe COVID-19 disease*	*192 (32.3)*	*173 (31.7)*	*19 (41.1)*
*I do not think new vaccines be efficient enough*	*208 (36.4)*	*196 (38.4)*	*12 (33.0)*
*I do not trust public authority about COVID-19 vaccination*	*196 (36.4)*	*177 (35.2)*	*19 (46.6)*
*I think vaccines serve the pharmaceutical industry*	*94 (18.1)*	*84 (16.1)*	*10 (34.9)*
**Have you downloaded the contact tracing mobile app (“TousAntiCovid”)? (y/n)**	503 (31.3)	412 (31.4)	91 (30.9)
**What are the sources you commonly use to inform yourself about COVID-19 vaccines?**			
Television news, radio, 24-h news channels …	1232 (88.4)	1028 (89.5)	204 (84.3)
Written press (daily press, digital newspapers …)	612 (44.0)	481 (41.6)	131 (52.5)
Social media (Facebook, Twitter, Instagram …)	807 (56.8)	673 (58.0)	134 (52.7)
Searching keywords on internet	235 (17.1)	177 (15.0)	58 (24.6)
Institutional website (vaccination-info-service …)	344 (25.5)	270 (23.5)	74 (32.5)
Recommendations (HAS, specialties college)	467 (35.9)	370 (33.9)	97 (43.1)
Scientific journals	242 (17.9)	180 (15.4)	62 (26.4)
University courses	242 (19.0)	191 (17.4)	51 (24.7)
I don’t look for information	57 (4.2)	49 (4.1)	8 (4.2)
**Some activities (trip, social events …) must be determined by the individual COVID-19 vaccination status**			
Agree	580 (38.1)	443 (34.0)	137 (52.7)
Neither agree or disagree	307 (20.5)	268 (21.8)	39 (15.8)
Disagree	558 (41.4)	489 (44.3)	69 (31.5)

^*^ %: proportions weighted on gender and training courses category

^†^ Students in the “common and first year of several health studies” are exclude because they do not do an internship

^‡^ (y/n): yes/no questions. For these questions, only the statistics for the “yes” modality are mentioned in the table

Univariate weighted odds ratio (wOR) and 95% confidence interval (95%CI) were estimated using logistic regression in the population (Table 3). Variables with *P* values < 0.2 in the univariate analysis were further assessed in the multivariable logistic regression model. A P value of < 0.05 was considered statistically significant to keep a variable in the multivariable model after a step by step decrease. Multivariable weighted odds ratio (awOR) and 95% CI were estimated using logistic regression. All statistical analyses were carried out using the software *R* in version 4.0 with the libraries *survey*, *svrepmisc* and *psy*. This study followed the Checklist for Reporting Results of Internet E-Surveys (CHERRIES) guidelines [13].

**Table 3:** Identifying factors associated with vaccine hesitancy towards COVID-19 vaccines among healthcare students. Weighted logistic regression analysis. COVID-19 vaccine hesitancy study among healthcare students, France, January–February 2021 (*n* = 1465)

	Vaccine hesitancy n (%)^*^		Univariate OR^†^ (95% CI)	***p***	Adjusted OR^‡^ (95% CI)
	Hesitancy ***N*** = 600	No hesitancy ***N*** = 862			
Gender					
Female	558 (89.3)	658 (68.8)	Ref		Ref
Male	42 (10.7)	204 (31.2)	0.27 (0.18, 0.39)	< 0.001	0.34 (0.19, 0.60)
Age					
< 19 years old	201 (30.6)	290 (30.9)	Ref		
[20–22] years old	248 (43.3)	373 (43.1)	1.01 (0.78, 1.32)	0.92	
> 23 years old	151 (26.0)	199 (26.0)	1.01 (0.74, 1.37)	0.95	
Training courses					
Common first year^§^	102 (10.5)	232 (21.4)	Ref		Ref
Medical (2nd year to 6th year)	56 (5.4)	292 (22.9)	0.48 (0.33, 0.70)	< 0.001	0.39 (0.23, 0.67)
Midwifery (2nd year to 5th year)	37 (5.1)	60 (6.8)	1.53 (0.95, 2.46)	0.08	0.79 (0.42, 1.50)
Physiotherapy (2nd year to 5th year)	237 (15.4)	104 (22.4)	1.41 (0.94, 2.10)	0.09	1.00 (0.57, 1.76)
Nursing (1st year to 3rd year)	64 (51.9)	109 (20.3)	5.20 (3.71, 7.28)	< 0.001	2.76 (1.70, 4.48)
Other	104 (11.7)	65 (6.3)	3.79 (2.56, 5.61)	< 0.001	1.70 (0.96, 3.03)
Internship during the epidemic^**^					
Yes	478 (86.6)	622 (77.5)	Ref		
No	122 (13.4)	240 (22.5)	0.53 (0.41, 0.69)	< 0.001	
Have presented COVID-19					
No	467 (75.5)	680 (78.3)	Ref		
Yes	133 (24.5)	182 (21.7)	1.17 (0.76, 1.21)	0.74	
Being contact case of a confirmed case at least once					
No	288 (46.4)	388 (45.4)	Ref		
Yes	312 (53.6)	474 (54.6)	0.96 (0.76, 1.21)	0.74	
Have had relatives ill and/or hospitalized for COVID					
None	205 (34.7)	239 (29.7)	Ref	0.72	
Relatives hospitalized for COVID	84 (14.6)	104 (11.7)	1.07 (0.74, 1.55)	0.02	
Relatives with COVID but not hospitalized	311 (50.8)	519 (58.7)	0.74 (0.57, 0.96)		
Having relatives at risk of severe COVID					
No	123 (21.1)	161 (18.0)	Ref		
Yes	477 (78.9)	701 (82.0)	0.82 (0.62, 1.10)	0.2	
Have downloaded the contact tracing mobile app					
No	474 (80.9)	486 (59.0)	Ref		Ref
Yes	126 (19.1)	376 (41.0)	0.34 (0.26, 0.44)	< 0.001	0.65 (0.44, 0.95)
Have been vaccinated against the 2020 seasonal flu					
No	520 (86.4)	545 (66.7)	Ref		Ref
Yes	80 (13.6)	317 (33.3)	0.32 (0.23, 0.42)	< 0.001	0.42 (0.27, 0.66)
Feeling or having felt isolated					
No	271 (48.7)	292 (38.1)	Ref		
Yes	304 (51.3)	535 (61.9)	0.65 (0.51, 0.82)	< 0.001	
Fear for one’s health					
No	465 (79.8)	719 (86.3)	Ref		
Yes	110 (20.2)	108 (13.7)	1.60 (1.16, 2.20)	0.004	
Fear for relatives’ health					
No	168 (28.1)	185 (22.7)	Ref		
Yes	407 (71.9)	642 (77.3)	0.75 (0.57, 0.98)	0.03	
Sources of information used^††^					
Do not look for information	31 (5.6)	26 (3.0)	Ref		
Use reliable sources	274 (47.8)	466 (57.1)	0.46 (0.25, 0.83)	0.01	
Use unreliable sources	281 (46.6)	365 (39.8)	0.64 (0.35, 1.16)	0.14	
Have personal good knowledge on COVID-19 vaccines					
Neither agree nor disagree	208 (35.4)	315 (36.5)	Ref		Ref
Agreed	88 (15.2)	270 (33.0)	0.47 (0.34, 0.66)	< 0.001	0.51 (0.30, 0.86)
Disagree	290 (49.4)	271 (30.5)	1.67 (1.29, 2.17)	< 0.001	1.14 (0.77, 1.69)
Act as a referral for family or friends to provide information about COVID-19					
Neither agree nor disagree	158 (26.9)	171 (20.5)	Ref		Ref
Agreed	180 (31.0)	433 (50.6)	0.47 (0.35, 0.63)	< 0.001	0.61 (0.39, 0.97)
Disagree	248 (42.1)	252 (28.9)	1.11 (0.82, 1.51)	0.5	0.96 (0.60, 1.55)
Consider that vaccination should be made mandatory for caregivers					
Neither agree nor disagree	159 (24.6)	212 (25.0)	Ref		Ref
Agreed	52 (8.2)	539 (62.5)	0.13 (0.09, 0.20)	< 0.001	0.33 (0.21, 0.53)
Disagree	375 (67.2)	105 (12.5)	5.46 (3.91, 7.63)	< 0.001	3.43 (2.17, 5.40)
Consider that healthcare students should participate to the vaccination campaign					
Neither agree nor disagree	232 (37.5)	192 (21.8)	Ref		Ref
Agreed	92 (16.2)	593 (70.6)	0.13 (0.10, 0.18)	< 0.001	0.31 (0.21, 0.47)
Disagree	262 (46.3)	71 (7.6)	3.55 (2.47, 5.09)	< 0.001	2.45 (1.47, 4.08)
Consider that certain activities should be conditioned by vaccine status					
Neither agree nor disagree	106 (17.1)	200 (23.1)	Ref		Ref
Agreed	89 (14.6)	491 (56.8)	0.35 (0.24, 0.49)	< 0.001	0.77 (0.49, 1.20)
Disagree	391 (68.3)	165 (20.1)	4.60 (3.32, 6.38)	< 0.001	1.73 (1.10, 2.73)

^*^ Weighted proportions. Amounts may be discreetly less than or greater than 100% due to rounding

^†^ Univariate odds-ratio (OR) are calculated using a weighted logistic regression

^‡^ Adjusted odds-ratio (aOR) are calculated using a weighted logistic regression

^§^ Common and first year of several health studies

^**^ This variable was not used in the multivariate model because the students of the common first year of medial, midwifery and physiotherapy studies do not carry out an internship during their training year

^††^ The variable was defined based on several responses about the nature of the sources of information used by the respondents. Thus, if the respondent mentioned looking for information on institutional sites (Ministry of health, recommendations from experts or medical associations …), in scientific journals, university lectures, it was indicated that he/she was using “reliable sources”. On the contrary, the respondent looking for information via mass media, general press, social networks, or the Internet was considered as using “unreliable sources”. Responses indicating that the respondent did not look for information at all were kept as such

### Ethics

The study was approved by the Research Ethics Committee of Paris Saclay University, Paris, France (Polethis) (CER-Paris-Saclay-2021-014) and the French data protection authority (Commission Nationale de l’informatique et des Libertés [CNIL]) (registration number: 2220726).

## Results

### Study sample

A total of 1465 healthcare students completed the online questionnaire, bringing the participation rate to 29.7% (Table 1). Amongst them, a large majority were female. Women outnumbered men by almost 5 to 1 (sex ratio = 4.96) in the sample, whereas they represented slightly less than 4 out of 5 students in the study population (sex ratio = 3.54). The mean age is 21.2 years (± 3.2); 33.5% of respondents are under 20 years old and 24.0% over 22 years old. Health studies in France are organized in distinct training courses but a first year of study is common to several of these (“common and first year of several health studies”) (Fig. 1). This first year of study is the same for the medicine, midwifery, and physiotherapy studies. In contrast, nursing studies and other health studies start with an independent first year. Thus, among the respondents, 334 (22.8%) declared that they were in the common and first year of several health studies. There were 342 (23.3%) students in the first year of nursing studies. There were also 349 (23.8%) students in medicine studies (2nd year or more), 174 (11.9%) students in physiotherapy studies (2nd year or more) and 97 (6.6%) students in midwifery studies (2nd year or more). The vast majority of the respondents (*n* = 1103) had done an internship during the epidemic period. During these internships, 58.5% of them took care of COVID-19 patients (Table 2, weighted percentage). Slightly more than one in four healthcare students (26.7%) said they had been vaccinated against influenza during the winter 2020–2021.

### Healthcare students experience of the COVID-19 pandemic

Nearly 15% of healthcare students reported having been infected with COVID-19 during the 2020 epidemic waves or in January 2021 (Table 2, weighted percentages). More than two thirds of them (68.1%) indicated that they had had cases of COVID-19 among their relatives. For 12.9% of them, their relatives were hospitalized for COVID-19. Concerning healthcare students’ feelings during the COVID-19 epidemic, 74.9% said they were worried about the health of their relatives, 71.0% said they had difficulty following the courses online, 57.2% felt isolated and 34.4% felt anxious. About one in 6 students (16.5%) declared they are concerned about their own health. Nearly one in three students (31.3%) reported having downloaded the contact-tracing mobile app entitled “TousAntiCovid”.

To inform themselves about COVID-19 vaccines, 88.4% of healthcare students used mass media such as television, radio or 24-h news channels. They were 56.8% to inform themselves on social networks and 44.0% using the written press (daily press, digital newspapers). On the other hand, only 19.0% of them said they got information about COVID-19 vaccines from their university courses and 17.9% from scientific journals (Table 2).

### Vaccine hesitancy and its determinants among healthcare students

**Fig. 2:**
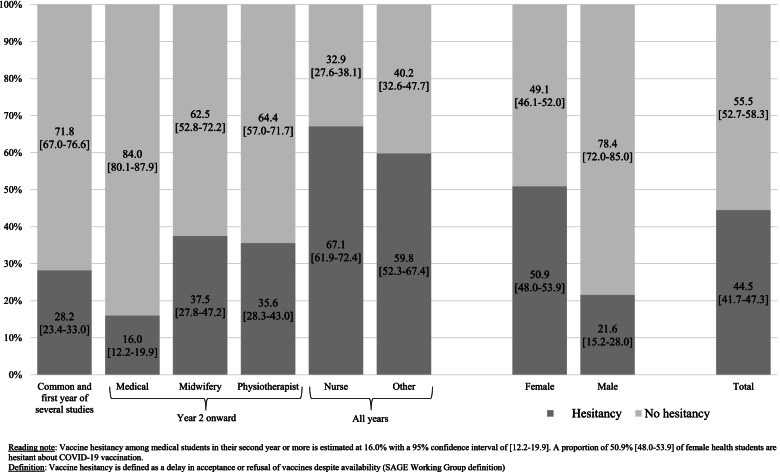
Acceptance and hesitancy towards COVID-19 vaccines. Weighted proportions after adjusting on gender and training courses, and 95% confidence intervals. COVID-19 vaccine hesitancy study among healthcare students, France, January–February 2021 (*n* = 1465)

When asked whether healthcare students would be willing to be vaccinated against COVID-19, 36.7% answered “yes, definitely” while 13.5% were certain not to be vaccinated (Table 2). According to the SAGE definition of vaccine hesitancy, 44.5% (95%CI = [41.7–47.3]) of the students could be considered as hesitant (Fig. 2, weighted percentages). Women were more hesitant (50.9%; 95%CI = [48.0–53.9]) than men (21.6%; 95%CI = [15.2–28.0]). Depending on the training courses, students were more or less hesitant about the COVID-19 vaccines. Thus, medical students (from the 2nd year onwards) were the least hesitant with 16.0% (95%CI = [12.2–19.9]) hesitant. Students in the common and first year of several health studies were 28.2% (95%CI = [23.4–33.0]) to declare themselves hesitant and 67.1% (95%CI = [61.9–72.4]) of nursing students were hesitant.

The reasons for vaccine acceptance and hesitancy were specified by the students (Table 2). Protection of their relatives was reported by 93.3% of them as a reason to get the vaccine. Eighty-eight percent (88.4%) wanted to return to their previous social life without restriction through vaccination and 84.6% wanted to be vaccinated to protect patients they take care of. Only slightly more than half of the students who said they wanted to be vaccinated believed in the safety and efficacy of the COVID-19 vaccines (52.8%). Vaccine safety appeared to be the main reason for healthcare students’ hesitation to vaccinate: 76.2% of them said they prefer to wait until the safety of the vaccines had been demonstrated and 54.7% feared side effects. Only 4.3% of students were against vaccines in general.

Vaccine hesitancy among healthcare students was significantly associated with gender: male were less likely to be hesitant than women (wOR = 0.27, 95%CI = [0.18–0.39], *p* < 0.001). Regarding training courses, medical students were less likely to be hesitant than students in the common and first year of several health studies (wOR = 0.48, 95%CI = [0.33–0.70], p < 0.001) while nursing students were more than 5 times more likely to be hesitant than the latter (wOR = 5.20, 95%CI = [3.71–7.28], p < 0.001). Students who did an internship during the epidemic (wOR = 0.53, 95%CI = [0.41–0.69], p < 0.001) and who downloaded the mobile contact-tracing mobile app (wOR = 0.34, 95%CI = [0.26–0.44], p < 0.001) were less likely to be hesitant.

In the adjusted model, vaccine hesitancy remained associated with gender (awOR = 0.34, 95%CI = [0.19–0.60]) and training courses (Table 3). Nursing students were almost 3 times more likely to be hesitant about the COVID-19 vaccination than the students in the common and first year of several health studies (awOR = 2.76; 95%CI = [1.70–4.48]). Downloading the contact-tracing mobile app (awOR = 0.65; 95%CI = [0.44–0.95]) and being vaccinated against the 2020 seasonal influenza virus (awOR = 0.42; 95%CI = [0.27–0.66]) were protective factors for vaccine hesitancy. Students who reported not having a good knowledge of vaccines were twice as likely to be hesitant (awOR = 2.23; 95%CI = [1.35–3.70]). Students who considered that certain activities should be conditional on being vaccinated had a 30% lower risk of being hesitant (awOR = 0.77; 95%CI = [0.49–1.20]) while those who said they were opposed to this statement had a 70% higher risk of being hesitant (awOR = 1.73; 95%CI = [1.10–2.73]). Respondents stating that healthcare students should participate in the vaccine campaign were less likely to be hesitant (awOR = 0.31; 95%CI = [0.21, 0.47]) than those who disagreed with this proposal (awOR = 2.45; 95%CI = [1.47, 4.08]).

## Discussion

In this study, vaccine hesitancy of healthcare students to the COVID-19 vaccination at the beginning of the year 2021 was estimated at 44.5, 95%CI = [41.7–47.3]. Vaccine hesitancy was significantly associated with gender, training courses and having downloaded the contact-tracing mobile app. It was also significantly associated with not being vaccinated against influenza, feeling or having felt isolated during the epidemic, and being afraid for their own health. Healthcare students who considered having a good knowledge of COVID-19 vaccines and that vaccination should be made mandatory for caregivers were significantly less likely to be reluctant to be vaccinated. Vaccine acceptance was finally associated with considering that healthcare students should participate in the vaccination campaign and, considering that certain activities should be conditional on being vaccinated.

Among the motivations for being vaccinated, healthcare students give priority to reasons related to protecting the health of their relatives or patients they might take care of. For a large majority of them, the perspective of a return to cultural, social, or sporting life and activity is an important motivation. Most healthcare students who are reluctant to be vaccinated say they have little confidence in the safety of the new vaccines and more than half of them fear side effects.

The use of the SAGE group definition of vaccine hesitancy allows comparisons between vaccine hesitancy of healthcare students in this study with those of other national and international studies [2]. Like that of the general population, vaccine hesitancy among healthcare students varies greatly from one country to another and from one continent to another [5]. A recent study among medical students in India, conducted between February and March 2021, showed that only 10.6% of the students were hesitant [14]. A survey of 2133 Egyptian medical students in January 2021 found that 46% were reluctant to be vaccinated against COVID-19 and nearly 20% refused outright [15]. In the French context, the COVIREIVAC survey conducted between May 10 and May 23, 2021, showed that 70% of French 18–24-year-olds surveyed were already vaccinated or said they wanted to be [16]. Nevertheless, these global estimates do not account for the great heterogeneity, according to the gender of the students and their training courses.

For example, it appears that women healthcare students are more hesitant than men healthcare students. This finding has been reported in studies conducted in the general population [7, 17] as well as in studies conducted among healthcare professionals [18]. We hypothesize that women are more cautious about new vaccines and typically about pregnancy concerns and fertility despite no proven relationship [19]. Indeed, they may be more concerned than men about potential side effects and vaccine safety. Our results show that among hesitant women and men, a higher proportion of women said they wanted to wait for new vaccines to be proven safe.

In our study, nursing students were 4 times more hesitant than medical students. This difference has already been demonstrated among healthcare workers [17, 20, 21], especially for vaccination against COVID-19, for which vaccination coverage varies according to the caregiver’s profession. At the end of July 2021, vaccination coverage for full vaccination was 70.6% among medical doctors, 56.7% among physiotherapists, 54.9% among nurses and 54.3% among midwives in France [22]. Similarly, our results show that midwifery students and physiotherapy students are approximately twice more hesitant than medical students. These observations are consistent with the differences in vaccine intention and coverage by healthcare profession for seasonal influenza [23]. The students would be more prone to follow the attitudes of their elders with whom they work during their internships. Indeed, the evolution of the vaccine hesitation rates between the common and first year of several health studies and the upper years of these studies suggests that the youngest students, who are not yet doing an internship in a professional environment, are relatively less hesitant than students who are further along in their training courses. Another hypothesis is that vaccine hesitancy among healthcare students could be partly explained by preconceived representations regarding vaccines in general. These representations could be addressed using interdisciplinary approaches for lectures on vaccine preventable diseases [24]. Prior to designing such educational programs on vaccination, exploring these representations with qualitative studies is needed, particularly among nursing students, whose reluctance to be vaccinated is significant. Such research could be conducted on a European scale to identify possible disparities between countries. It is also important to ask whether, within each of the European countries, differences in terms of vaccine hesitancy exist in the same proportions or not. Recent European studies have shown that vaccine hesitancy is not different between healthcare students and other students [25]. The specificity of healthcare students could thus be questioned in a broader perspective by considering the role and place occupied by the healthcare professions in the different European health systems.

Our results also show that healthcare students are widely solicited by their entourage, probably because of the nature of their studies, which places them as a reliable source of information on health issues. They are also able to convey prevention messages for their relatives, as it has already been shown in other studies [26, 27]. In this context, it is particularly reassuring to see healthcare students being proactive about the epidemic: downloading the contact-tracing mobile app “TousAntiCovid” [28] or seeking information from reliable sources as other studies have already shown [29]. In this way, healthcare students could help limit the spread of misinformation within their age group and in their families.

Our study also asked healthcare students about the impact of COVID-19 and vaccination on their personal lives. As with many students, they reported feeling particularly isolated during the various epidemic waves, and in particular during the first two waves that marked the year 2020 [30]. In this study, they expressed a desire to return to a more fulfilling social life, with a majority of them saying they were in favor of a covid-health pass. Similarly, at the beginning of 2021, the majority of healthcare students were in favor of the mandatory vaccination of healthcare professionals, which will ultimately be effective since 15 September 2021.

As this study cover the beginning of 2021, it could be repeated in order to monitor the evolution of healthcare students’ perceptions and attitudes towards COVID-19 vaccination, especially since regulatory measures (covid-health pass, vaccination mandatory for healthcare professionals) have been taken to accelerate vaccination campaign in France. Indeed, vaccination has been officially extended to younger people without comorbidities since May 31, 2021. At this time, vaccine coverage was estimated at 19.9% (one or two doses) and reached 48.5% July 10, 2021 prior to the announcement of the implementation of the covid-health pass for those over 18 starting from August 9, 2021. Thereafter, vaccine coverage for 18–24-year-olds (one or two doses) increased to 60% at the end of July 2021 to achieve 84% at the end of August [31]. The significant increase in vaccination coverage of young people before the introduction of the covid-health pass would highlight the variety of factors that may explain vaccine hesitancy in this age group and reinforces the need to study vaccine hesitancy through its determinants, especially since the covid-health pass is intended to be used only in a transitory manner. Considering COVID-19 vaccine hesitancy through its determinants should allow to implement vaccination promotion strategies based on health education approaches, especially that some factors associated with COVID-19 vaccine hesitancy are also found for other vaccines as well. This is all the more important as these challenges also concerned other European countries [5, 25] and thus need effort at the European level.

Although our study sample represented only 30% of the study population, all the estimates, including vaccine hesitancy and measures of association, were a posteriori weighted on gender and training courses category. As the study was carried out at the very beginning of the implementation of the vaccination campaign in France and over a very short period of time (only 18 days), we consider that the responses of the participants in the survey could not have been influenced by the information and vaccination promotion campaigns which remained the same during the survey period. In addition, young people were not primarily concerned by vaccination because they were not among the priority people.

It is important to remain cautious about the generalizability of our results to the entire population of healthcare students in France, as this study was not conducted in all the universities. However, it allowed us to question students from different training courses and to consider comparisons between these training courses. The choice of a non-probabilistic sampling exposes us to a selection bias which we have nevertheless tried to avoid by proceeding with a weighting to produce adjusted proportions on our population. In addition, because of the social desirability bias, the estimate of vaccine hesitancy among healthcare students may have been underestimated. This bias may also have influenced the estimates of the measures of association. However, we believe we reduced the magnitude of this bias by conducting an Internet questionnaire rather than a face-to-face one. Despite these potential limitations, we think that this study provides reliable estimates about vaccine hesitancy and its determinants among healthcare students.

## Conclusions

Overall vaccine hesitancy among healthcare students was high. To get insight into determinants of vaccine hesitancy in healthcare students could be essential in the perspective of a long-term COVID-19 epidemic and the possible need for subsequent vaccination campaigns including a possible third dose or seasonal vaccine. In addition, substantial differences in vaccine hesitancy were found between training courses. To reduce these disparities, dedicated interdisciplinary lectures on vaccines for all healthcare students at the University may be implemented and evaluated.

## Supplementary Information

**Additional file 1.**

## Data Availability

The European Data Protection Regulation (GDPR) provides a clear framework for international data transfers. Data transfers, especially outside the EU, must be assessed on a case-by-case basis in accordance with Article 45 of the GDPR. In accordance with this regulation, the datasets used and/or analyzed during the current study are available from the corresponding author on reasonable request.

## Abbreviations

awOR: Adjusted weighted odds ratio
CHERRIES: Checklist for Reporting Results of Internet E-Surveys
CNIL: Commission Nationale de l’informatique et des Libertés
COVID-19: Coronavirus disease-2019
SAGE: Strategic Advisory Group of Experts on Immunization
USA: United States of America
WHO: World Health Organization
wOR: Weighted odds ratio
