# Supplementary material for: Cross-sectional study on COVID-19 vaccine hesitancy and determinants in healthcare students: interdisciplinary trainings on vaccination are needed
Source: BMC Med Educ. 2022 Apr 20;22:299. doi: 10.1186/s12909-022-03343-5 (PMC9020813; doi:10.1186/s12909-022-03343-5)
Supplement: Supplementary file 1 — Additional file 1. [file 12909_2022_3343_MOESM1_ESM.pdf]

The purpose of this questionnaire is to document the perception of the COVID-19 vaccination among health students at the UFR Simone Veil-Santé (UVSQ) as well as their attitude towards this vaccination.

This research study is conducted by the epidemiology and public health department of the Raymond Poincaré hospital in Garches.

You must be at least 18 years old to participate in this study. By answering the questionnaire, you agree to participate in this research study. Your answers are entirely anonymous and their statistical processing will be the subject of scientific publications.

Answering the questionnaire will take you about ten minutes. There are no "right" or "wrong" answers, it's your opinion that counts above all.

Your participation is very important!

By completing this questionnaire, you agree that the data provided will be used for research purposes. The questionnaire being completely anonymous, no request for access to your data, rectification or erasure can be taken into account after having completed and validated the questionnaire because it is impossible for us to identify you or to link you to your questionnaire.

CNIL number: pending.

A1. Have you already answered this survey?

- Yes ☐
- Nope ☐

B1. Are you :

- A man ☐
- A woman ☐

B2. How old are you ?

- <18 years old ☐
- 18 years old ☐
- 19 years old ☐
- 20 years ☐
- 21 years old ☐
- 22 years old ☐
- 23 years ☐
- 24 years ☐
- 25 years ☐
- 26 years ☐
- 27 years old ☐
- 28 years ☐
- 29 years ☐
- 30 years ☐
- >30 years ☐

B3. **What is your registration program at the UFR Simone Veil-Santé (UVSQ)?**

- PASSES/SPACES
- Medicine (excluding PASS/PACES)
- Maieutics
- Nurse / APN
- Other sector (physiotherapy, occupational therapy, psychomotricity, podiatry, manipulation in medical electroradiology)
- Health Masters, DU-DIU
- I am not a student at UFR Simone Veil-Santé of UVSQ

B4. **What is your year of study?**

2nd year of medicine (DFGSM2) 3rd year
of medicine (DFGSM3) 4th year of
medicine (DFASM1) 5th year of medicine
(DFASM2) 6th year of medicine (DFASM3)
3rd cycle: general medicine internship 3rd
cycle: other internship specialty

B5. **What is your year of study?**

2nd year of midwifery 3rd
year of midwifery 4th year of
midwifery 5th year of midwifery

B6. **What is your year of study?**

1st year nurse 2nd year
nurse 3rd year nurse 1st
year advanced practice
nurse 2nd year advanced practice nurse

B7. **What is your year of study?**

1st year of physiotherapy 2nd
year of physiotherapy 3rd year of
physiotherapy 4th year of
physiotherapy 1st year of
occupational therapy 2nd
year of occupational therapy
3rd year of occupational
therapy 1st year of psychomotricity
2nd year of psychomotricity 3rd
year of psychomotricity 1st year of
pedicure-podiatry 2nd year of pedicure-
podiatry 3rd year of pedicure-podiatry
1st year of medical electroradiology
manipulation 2nd year of medical electroradiology manipulation
3rd year of medical electroradiology manipulation

B8. **What is your year of study?**

- M1
- M2
- DU-DIU

B9. What service(s)/internship(s) have you been in since the beginning of the COVID epidemic? (One or more possible answer(s))

I did not go to any Emergency Infectious
Diseases
department
Intensive care
Geriatrics
Internal Medicine
Pulmonology
General medicine practice
Other service(s)/internship(s)

B10. In this (these) service(s)/internship(s), did you have to deal with patients with COVID-19?

- Yes ☐
- Nope ☐

B11. Have you been requisitioned, reassigned or have you responded to a request for mobilization (from health authorities or establishments)?

- Yes ☐
- Nope ☐

C1. Have you had COVID-19?

- Yes, with diagnosis confirmed by a positive test ☐
- Yes, I'm sure but I haven't done a test. ☐
- No I do not know ☐

C2. Have you had any cases of COVID-19 in your entourage (close family, friends)?

- Yes ☐
- Nope ☐
- I do not know ☐

C3. Have you had any of your loved one(s) hospitalized with COVID-19?

- Yes, hospitalization in the medical department (excluding intensive care) ☐
- Yes, intensive care hospitalization ☐
- Nope ☐

C4. Do you consider yourself to belong to the population "at risk" of severe forms of COVID-19?

- Yes, because I have comorbidities ☐
- Yes, because I am exposed as a health student ☐
- Yes, for another reason ☐
- Nope ☐
- I do not know ☐

C5. Do you have relatives (family, friends) considered to belong to the "population at risk" of severe forms of COVID-19?

According to the HAS: >65 years old, complicated arterial hypertension, heart failure, history of stroke, coronary heart disease or heart surgery, unbalanced or complicated diabetes, obesity with BMI >=30, chronic respiratory pathology, progressive cancer under treatment, chronic renal failure dialysis, pregnant woman with comorbidity or in the 3rd trimester of pregnancy…

- Yes ☐
- Nope ☐
- I do not know ☐

C6. Regarding your experience of the epidemic, with which statements do you agree? (One or more possible answer(s))

- I lived the 1st period of confinement well ☐
- I lived well the 2nd period of confinement ☐
- I felt isolated ☐
- I was very upset ☐
- I feared for my health ☐
- I feared for the health of my loved ones ☐
- Following the courses remotely was complicated ☐

C7. Have you downloaded the "TousAntiCovid" (or "StopCovid") application on your smartphone?

- Yes ☐
- Nope ☐
- I have never heard of this app ☐

D1. Are you up to date with your vaccination schedule?

- Yes ☐
- Nope ☐
- I do not know ☐

D2. Have you been vaccinated this winter (2020) against the flu?

- Yes ☐
- No, but I intend to ☐
- No, I don't intend to ☐

D3. Have you been vaccinated against human ***papillomavirus*** infection **(cervical cancer vaccine)?**

- Yes ☐
- No, my parents were not in favor of it ☐
- No, my pediatrician/general practitioner did not recommend it ☐
- No, for another reason ☐
- I do not know ☐

D4. About the following statements would you say that you are: 1: completely disagree, 2: disagree, 3: neither agree nor disagree, 4: agree, 5: completely agree:

	1: totally disagree	2: disagree	3: neither okay neither disagree 4: agree		5: totally agree
Vaccines are useful to protect populations at risk	☐	☐	☐	☐	☐
Vaccines are useful to protect (oneself)	☐	☐	☐	☐	☐
There is a collective interest in vaccination	☐	☐	☐	☐	☐
Vaccines have eradicated some infectious diseases	☐	☐	☐	☐	☐
There is too great a risk of vaccines compared to the benefits provided	☐	☐	☐	☐	☐
I get vaccinated when my doctor recommends it	☐	☐	☐	☐	☐
I only get vaccinated if it is compulsory for students/health professionals	☐	☐	☐	☐	☐

E1. Would you be willing to get vaccinated against COVID-19?

- Certainly yes ☐
- Yes, probably ☐
- Perhaps ☐
- No, probably not ☐
- No, definitely not ☐

E2. What are the reasons why you are ready to get vaccinated against COVID-19? (One or more possible answer(s))

- Protect myself against COVID-19 ☐
- Protect my loved ones from COVID-19 ☐
- Avoid transmitting COVID-19 to patients ☐
- Contribute to controlling the epidemic ☐
- Resume my social, cultural and sporting life... ☐
- Available vaccines are generally effective and safe according to scientific studies ☐
- Other reason ☐

E3. What are the reasons you are not ready to get vaccinated against COVID-19? (one or more possible answer(s))

- I prefer other means to protect myself, such as barrier gestures or wearing a mask ☐
- I prefer to wait until the safety of new vaccines is proven to get vaccinated ☐
- I'm afraid of side effects ☐
- I am against vaccination in general ☐
- I already had COVID-19 ☐
- I am not at risk of severe form of COVID-19 ☐
- I don't think the new vaccines are effective enough ☐
- I don't trust government messages on vaccination ☐
- I think vaccines are used to enrich the pharmaceutical industries ☐
- Other reason ☐

E4. On a scale of 0 to 5, how confident are you with the COVID-19 vaccines that are or will be available in Europe? Give a score between 0 (not at all confident) and 5 (very confident).

- ☐ 0 – not at all confident
- ☐ 1
- ☐ 2
- ☐ 3
- ☐ 4
- ☐ 5 – very confident

E5. Where did you get information about COVID-19 vaccines? (One or more possible answers)

- ☐ Media (television news, radio, continuous news channel, etc.)
- ☐ Press (paper daily newspapers, digital newspapers, popular science magazines, etc.)
- ☐ Social networks (Facebook, Twitter, Instagram…)
- ☐ Search by keywords on the internet (search engine)
- ☐ Institutional sites (vaccination-info-service…)
- ☐ Recommendations (HAS, specialty colleges)
- ☐ Scientific journals
- ☐ University courses
- ☐ I'm not looking for information

E6. Regarding the following statements, would you say that you are: 1: totally disagree, 2: disagree, 3: neither agree nor disagree, 4: agree, 5: totally Okay.

	1: totally disagree	2: disagree	3: neither agree nor disagree	4: agree	5: absolutely Okay
I have good knowledge about COVID-19 vaccines	☐	☐	☐	☐	☐
I am requested by those around me to relay information on COVID-19	☐	☐	☐	☐	☐
Vaccination against COVID-19 must be made compulsory for healthcare workers	☐	☐	☐	☐	☐
Healthy students must participate in the vaccination campaign against COVID-19	☐	☐	☐	☐	☐

Thank you for answering our Hévéa-cov questionnaire on the perception of COVID-19 vaccine by healthy students!
